# Prediction of Ecofriendly Concrete Compressive Strength Using Gradient Boosting Regression Tree Combined with GridSearchCV Hyperparameter-Optimization Techniques

**DOI:** 10.3390/ma15217432

**Published:** 2022-10-23

**Authors:** Zaineb M. Alhakeem, Yasir Mohammed Jebur, Sadiq N. Henedy, Hamza Imran, Luís F. A. Bernardo, Hussein M. Hussein

**Affiliations:** 1 Computer Engineering Department, Iraq University College, Basrah 61004, Iraq; 2 Building and Construction Techniques Engineering Department, Al-Mustaqbal University College, Hillah 51001, Iraq; 3 Department of Civil Engineering, Mazaya University College, Nasiriya City 64001, Iraq; 4 Department of Environmental Science, College of Energy and Environmental Science, Alkarkh University of Science, Baghdad 10081, Iraq; 5 Centre of Materials and Building Technologies (C-MADE), Department of Civil Engineering and Architecture, University of Beira Interior, 6201-001 Covilhã, Portugal; 6 Medical Physics Department, Hilla University College, Babylon 51002, Iraq

**Keywords:** gradient boosting regression tree, machine learning, SHAP, eco-friendly concrete, compressive strength, prediction

## Abstract

A crucial factor in the efficient design of concrete sustainable buildings is the compressive strength (Cs) of eco-friendly concrete. In this work, a hybrid model of Gradient Boosting Regression Tree (GBRT) with grid search cross-validation (GridSearchCV) optimization technique was used to predict the compressive strength, which allowed us to increase the precision of the prediction models. In addition, to build the proposed models, 164 experiments on eco-friendly concrete compressive strength were gathered for previous researches. The dataset included the water/binder ratio (W/B), curing time (age), the recycled aggregate percentage from the total aggregate in the mixture (RA%), ground granulated blast-furnace slag (GGBFS) material percentage from the total binder used in the mixture (GGBFS%), and superplasticizer (kg). The root mean square error (RMSE) and coefficient of determination (R^2^) between the observed and forecast strengths were used to evaluate the accuracy of the predictive models. The obtained results indicated that—when compared to the default GBRT model—the GridSearchCV approach can capture more hyperparameters for the GBRT prediction model. Furthermore, the robustness and generalization of the GSC-GBRT model produced notable results, with RMSE and R^2^ values (for the testing phase) of 2.3214 and 0.9612, respectively. The outcomes proved that the suggested GSC-GBRT model is advantageous. Additionally, the significance and contribution of the input factors that affect the compressive strength were explained using the Shapley additive explanation (SHAP) approach.

## 1. Introduction

In recent years, the design and construction of sustainable buildings has become a major goal. One of the most important factors influencing this criterion is the use of Portland cement substitutes. For instance, cement production consumes vast energy and significantly contributes to greenhouse gas emissions and environmental degradation. Therefore, a reduction in cement incorporated in concrete mixes is necessary to protect the environment. In addition, to avoid the negative impacts of burying hazardous industrial wastes, using recycled concrete aggregate (RCA) and ground granulated blast-furnace slag (GGBFS) as waste materials in concrete mixes would also allow us to reduce greenhouse gas emissions, since less cement and natural aggregate (NA) will be produced [1].

Some researchers investigated the behavior of RCA in terms of durability and mechanical performance. They found that RCA has lower properties than natural concrete aggregate (NCA) [2,3,4,5,6,7].

Hence, concrete scientists must increase the qualities of RCA in order to improve the products produced with it due to the numerous environmental advantages of using RCA in the construction sector. Additionally, improving RCA makes it possible to utilize it to prepare concrete for a structure incorporating load-bearing structural members. However, old connected mortar, crushing techniques, and a poor interfacial transition zone (ITZ) are the critical causes for RCA’s inferior quality. Previous studies have shown that whereas RCA has five phases—NA, old ITZ, old cement paste, new ITZ, and new cement paste—NCA has three phases, which are NA, ITZ, and cement mixture. As a result, the rheological, mechanical, and durability characteristics of RCA are gradually decreased because of the higher significant porosity and water absorption capacity caused by higher number of phases. Various approaches have been reported in the literature to reduce the lower properties of RCA and make it similar to NCA. Incorporating supplemental cement-based materials such as ground granulated blast-furnace slag (GGBFS) was one of the solutions to improve the RCA properties and eliminate its lower properties [8].

Slag cement, commonly known as GGBFS, is a by-product of the iron production sector. A hydraulic cementitious substance with pozzolanic properties is a slag cement. Any reactive aluminosilicate substance is referred to as pozzolan. Pozzolans interact with the cement hydration process by-products to generate concrete. Pozzolans do not often provide enough strength when combined with water alone; thus, cement must be added to the mixture. Because of this, it is widely believed in the concrete industry that cement, at the very least in a reasonable proportion, is necessary to provide the necessary strength. When combined with water, slag cement, which has cementitious qualities similar to cement, will hydrate and provide strength.

Studies on the impact of GGBFS on several RCA properties have been performed. When comparing with NCA, Çakır [9] reported that concrete with 30% GGBFS and 50% RCA shows good mechanical properties. Similarly, according to Majhi and Nayak [10], concrete containing 40% GGBFS and 50% RCA has good mechanical and physical properties. It was also found that this concrete is as durable as NCA. Rashad [11] looked into how GGBFS content affects the compressive strength (Cs) and found that a mix with 50% GGBFS can have similar characteristics as concrete mix with ordinary Portland cement (OPC) content. In their study on the effects of 65% GGBFS on the properties of RCA, Ann et al. [12] discovered that GGBFS enhances the resistance of RCA to permeability and corrosion when used with 100% RCA. Afroughsabet et al. [13] studied the impact of adding 30% GGBFS to RCA. They found that substituting 30% OPC with GGBFS increased both the concrete splitting tensile and flexural strength while maintaining its Cs. Slag was shown to reduce both the water absorption and workability of concrete. RC beams using recycled aggregate from electric arc furnaces (EAF) were the subject of a study to determine how they would behave [14]. According to the experimental results, EAF concrete beams have enhanced material qualities, resulting in higher ultimate flexural and shear capacities and smaller crack widths than equivalent standard RC beams. Another study [15] concluded that utilizing ternary cements, which have the right proportions of blast-furnace slag and limestone filler, can minimize gaseous emissions without sacrificing the cement mechanical qualities and promote the effective use of by-products and natural resources.

One of the most crucial aspects to consider while building and engineering structures is testing the concrete Cs. Typically, the compression test for the specimens at the specified curing age is used in the lab to determine the Cs. However, in addition to time and money, a significant amount of material is needed to carry out this test. As a result, some researchers have developed a technique to estimate the Cs of eco-friendly concrete utilizing RAC as a replacement material for natural aggregate or GGBFS as a replacement material for ordinary cement.

## 2. Related Works

In civil engineering, supervised machine learning models have acquired a lot of momentum, especially for predicting eco-friendly concrete properties, because they can forecast the results with high accuracy. Researchers have presented many machine learning (ML)-based prediction models for forecasting the mechanical and physical properties of RCA. The suggested approaches have been validated as a reliable alternative to expensive and time-consuming testing in laboratory usually used to assess concrete qualities. For example, Han at al. [16] presented an ensemble ML model for concrete derived from RCA to estimate the concrete modulus of elasticity. It is demonstrated that when compared to stand-alone models, the ensemble ML model consistently generates more precise predictions. Additionally, prediction models for the Cs of concrete incorporating recycled materials were suggested using random forest, linear regression, and nonlinear regression techniques [17]. The models based on random forest and nonlinear regression were found to be more accurate than linear regression. Furthermore, Liu et al. [18] used ML algorithms to predict the carbonation depth in RCA. According to the results, the random forest model outperforms the stand-alone artificial neural network (ANN) model and Gaussian progress regression model. Last but not least, 10 ML algorithms were tested using a dataset containing 962 experimental Cs of NCA and RCA [19]. As they outperform existing methods, the ML models established for this work can be suggested as a valuable tool for the prediction of the Cs. In term of GGBFS, numerous studies established an ML model to forecast the mechanical characteristics of eco-friendly concrete that includes GGBFS material as a substitute for regular cement. The NN and ANFIS models for predicting chloride permeability in concrete were introduced by Boğa et al. [20]. Their study used concrete specimens containing solely calcium nitrite-based corrosion inhibitor (CNI), GGBFS, and a mixture of these ingredients in various ratios. The evaluation of the results demonstrated that both models estimate the permeability of chloride with good precision. In addition, random forest was used by Mai et al. [21] to forecast the Cs of GGBFS-containing concrete. RF performances in terms of R^2^, RMSE, and MAE were 0.9729, 4.9585, and 3.9423, respectively. Han et al. [22] created an innovative hybrid model for calculating the Cs of GGBFS concrete and validated the synergistic benefits of the hybrid algorithm over a single algorithm. The new PSO-BP hybrid neural network model outperformed basic ANNs trained by a single method and was shown to be suited for estimating the Cs of GGBFS concrete. Table 1 provides a comprehensive summary of relevant prior work related to predicting the Cs of eco-friendly concrete.

## 3. Research Significance

A powerful ensemble learning approach built on a gradient boosting system is called a gradient boosting regression tree (GBRT) [31]. To be more precise, GBRT is a robust data-mining technique that has been extensively tested and shown to be successful in various classification and regression problems [32,33,34,35,36,37,38,39,40,41,42,43]. As a result, in this study the prediction of the Cs of eco-friendly concrete was chosen to demonstrate the potential of the GBRT technique. Additionally, adjusting the hyperparameters of GBRT models for eco-friendly concrete datasets is beneficial. An essential step in the ML process is hyperparameter tuning (optimization). A wise selection of hyperparameters may either help a model to achieve the intended metric value or, on the other hand, cause it to enter an endless loop of iterative training and optimization. Thus, the hyperparameters of GBRT are optimized using the GridSearchCV technique. Since the prediction of the Cs of eco-friendly concrete that contains recycled aggregate (RA) as a replacement for NA and GGBFS material as a replacement for OPC has never been studied in the way that this study does, this is a novel piece of research. This article has the following format. The research technique for this work is outlined in Section 2, and Section 3 displays a description of the dataset. The outcomes from the model prediction and the comparative analysis are described in Section 4. Section 5 provides a thorough analysis of the significance and contribution of each input variable to the final Cs. Finally, Section 6 provides a summary of the main conclusion.

## 4. Materials and Methods

### 4.1. Research Methodology

**Figure 1:**
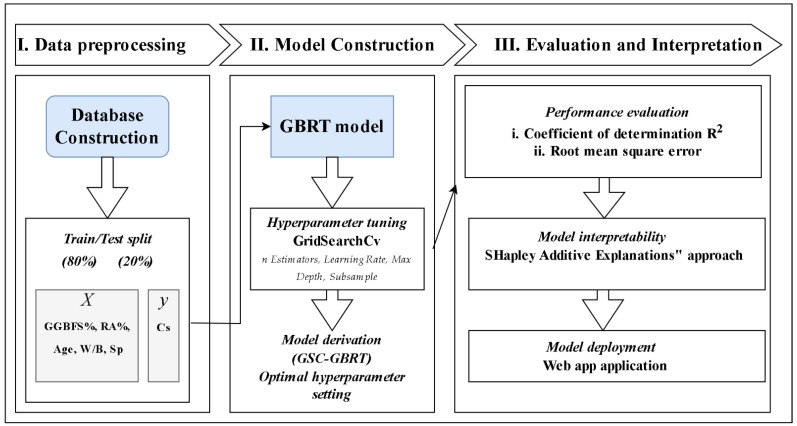
Research methodology.

The methodology of the study is displayed in Figure 1. The data were first gathered, then divided into training and testing datasets: 80% and 20% of the total data, respectively. The GBRT technique for generating models and the GridSearchCV approach for determining precise model parameters were then introduced. The evaluation and interpretation of the GSC-GBRT model made up the last phase. A performance assessment was carried out based on the statistical performance measurement metrics. Related metrics were used to quantify the evaluation results, including RMSE and R^2^ measurements. The notion of SHAP was finally introduced throughout the interpretation process, and global and local assessments were carried out. The Python 3.7 Scikit-learn software [44] was used to model and tune the GBRT in order to produce GSC-GBRT.

### 4.2. Gradient Boosting Algorithm

Let us consider *x* as a collection of random inputs variable $x=\left\{x_{1},x_{2},\ldots ,x_{n}\right\}$ and y as response variable. Using a training data in the form of $\left\{\left(x_{i},y_{i}\right)\right\}$ for i=1,2,…,N with $x_{i}\in R^{n}$ and $y_{i}\in R$, the goal is finding an approximation $\tilde{F}\left(x\right)$ of the function *F(x)* mapping x to y, to minimize loss function L(y,F(x)), see Equation (1). Errors are inevitable when it is expected to seek function $\tilde{F}\left(x\right)$. Each weak learner model seeks to correct errors produced by earlier weak learner models as the gradient boosting approach fits weak learners to the loss functions. Because of this, the performance of the prediction model may be improved, and prediction error can be decreased.

(1) $$\tilde{F}\left(x\right)={\;\mathrm{argmin}}_{F\left(x\right)}L_{y,x}\left(y,F\left(x\right)\right)$$

The estimation of the approximation function as $L\left(y,F\left(x\right)\right)={\left(y-F\left(x\right)\right)}^{2}$ needs the squared error function to be used as the loss function. The gradient boosting technique uses the steepest descent step to minimize the loss function after establishing an initial base learner $F_{0}\left(x\right)$, which is typically a constant function in step 1. Step 2 requires defining the iteration space for m = 1, …, M. Finding the local minimum requires the steepest descent, which makes steps proportional to the negative gradient of the loss function.

Specifically, the following equation can be used to determine the gradient of the loss function L(y,F(x)) (step 3):

(2) $${\hat{y}}_{i}=-{\left[\frac{\partial L\left(y_{i},F\left(x_{i}\right)\right)}{\partial F\left(x_{i}\right)}\right]}_{F\left(x\right)=F_{m-1}\left(x\right)},\;i=1,\cdots ,N$$

When regression trees $h\left(x_{i};a\right)$ are employed with parameter a as weak learners, it can generalize the range of the gradient calculation. The a parameters define it as a parameterized function of the input variables x [45]. The equation below can be solved to produce the tree (step 4):

(3) $$a_{m}={\;\mathrm{argmin}}_{a,\beta }\overset{N}{\underset{i=1}{\sum }}{\left[{\hat{y}}_{i}-\beta h\left(x_{i};a\right)\right]}^{2}$$

where β is the weight value, commonly known as the expansion coefficient of each weak learner, and $a_{m}$ is the parameters discovered at iteration m. The current negative gradient is fitted to each regression tree. The model $F_{m}\left(x\right)$ is then updated at step 6 at each iteration m, with m = 1, …, M, after step 5 determines the ideal length $p_{m}$. Algorithm 1 from Friedman formalizes the gradient boosting algorithm [29], see Algorithm 1.
**Algorithm 1:** gradient boosting.1: $F_{0}\left(x\right)=\underset{\rho }{\arg\min}\sum_{i=1}^{N}L\left(y_{i},\rho \right)$2: For m=1 to *M* do;3: ${\tilde{y}}_{i}=-{\left[\frac{\partial L\left(y_{i},F\left(x_{i}\right)\right)}{\partial F\left(x_{i}\right)}\right]}_{F\left(x\right)=F_{-1}\left(x\right)}$, i=1,…,N4: $a_{m}=\underset{a,\beta }{\arg\min}\sum_{i=1}^{N}{\left[{\tilde{y}}_{i}-\beta h\left(x_{i};a\right)\right]}^{2}$5: $\rho_{m}=\underset{\rho }{\arg\min}\sum_{i=1}^{N}L\left(y_{i}-F_{m-1}\left(x_{i}\right)+\rho h\left(x_{i};a_{m}\right)\right)$6: $F_{m}\left(x\right)=F_{m-1}\left(x\right)+\rho_{m}h\left(x;a_{m}\right)$7: End for8: End algorithm


### 4.3. Gradient Boosting Regression Tree Algorithm (GBRT)

Lie et al. [46] introduced classification and regression trees (CARTs) in 1984. CARTs can be used for regression and classification models [31,32,33,34,35]. The trees utilized in these two models are known as decision trees, and the development of decision trees involves using recursive techniques to produce binary trees. The technique that creates regression trees using the square error minimization criteria is mainly discussed here since the aim is to research concrete Cs predictions. It is noted that He et al. [47] introduced the GBRT method, which combines the CART algorithm with the GB algorithm. Because it can represent nonlinear interactions without requiring previous knowledge of the probability distribution of variables, it is noted that the CART has highest excellent prediction performance than most artificial intelligence models [47]. As mentioned before, the gradient boosting algorithm combines weak and robust learners. Regression trees produced by the CART method serve as weak learners in this investigation. In order to further minimize the prediction error and raise the model accuracy, the weak learners are added to the model to correct the prediction errors created by previous models.

The formalization of GBRT Algorithm 2 is presented in Algorithm 2.
**Algorithm 2:** GBRT.1:$F_{0}\left(x\right)=\underset{c}{\arg\min}\sum_{i=1}^{N}L\left(y_{i},c\right)$2: m=1 to *M* do;3: $r_{m,i}=-{\left[\frac{\partial L\left(y_{i},F\left(x_{i}\right)\right)}{\partial F\left(x\right)}\right]}_{F\left(x\right)=F_{m-1}\left(x\right)},i=1,\ldots ,N$4: $c_{m,j}=\underset{c}{\arg m}\underset{x_{i}\in R_{m,j}}{\sum }L\left(y_{i},F_{m-1}\left(x\right)+c\right)$5: $F_{m}\left(x\right)=F_{m-1}\left(x\right)+\sum_{j=1}^{J_{m}}c_{m,j}I\left(x\in R_{m,j}\right)$6: $F_{M}\left(x\right)=F_{0}\left(x\right)+\sum_{m=1}^{M}\sum_{j=1}^{J_{m}}c_{m,j}I\left(x\in R_{m,j}\right)$7: End for8: End algorithm


The initial value of *F*_0_(*x*) is set by the GBRT algorithm using the following equation (step 1).

(4) $$F_{0}(x)={\mathrm{argmin}}_{c}\sum_{i=1}^{N}L\left(y_{i},\rho_{0}\right)$$

*L*(.) in the previous equation is a loss function. The following equation is used to determine the value of the current loss function negative gradient in the model for the residual approximation at iteration *m*, where *m* = 1, 2, …, *M*. (step 3).

(5) $$r_{m,i}=-{\left[\frac{\partial L\left(y_{i},F\left(x_{i}\right)\right)}{\partial F\left(x_{i}\right)}\right]}_{F\left(x\right)=F_{m-1}\left(x\right)},i=1,\cdots ,N.$$

Each regression tree divides the input space $J_{m}$ into disjoint regions $R_{m,1}$, …, $R_{m,Jm}$ and predicts a value $c_{m,j}$ for region $R_{m,J}$ based on the assumption that there are $J_{m}$ splits. The following equation can be minimized to get the value of $c_{m,j}$ (step 4).

(6) $$c_{m,j}=\underset{c}{\mathrm{argm}}\sum_{x_{i}\varepsilon R_{m,j}}L(y_{i},F_{m-1}(x)+c)$$

The updated model, or the *m-*th regression tree *F*_m_(*x*), whose corresponding leaf node area is $R_{m,J}$ *j* = 1, 2, …, *J*_m_, may be calculated as follows (step 5).

(7) $$F_{m}\left(x\right)=F_{m-1}\left(x\right)+\sum_{j=1}^{J_{m}}c_{m,j}I\left(x\in R_{m,j}\right)$$

where $J_{m}$ is a representation of the *m-*th regression tree number of leaf nodes and I=1 if *x* ∈ $R_{m,J}$ and I = 0 otherwise. At step 6, the model is lastly updated.

### 4.4. Hyperparameter Tunning with GridSearchCV

**Figure 2:**
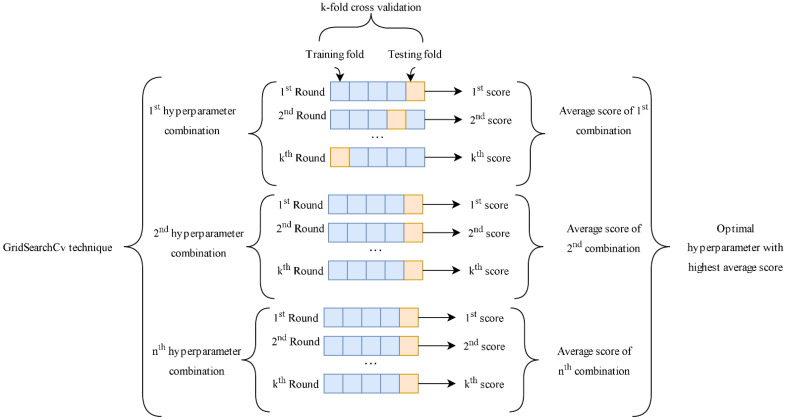
Tuning of hyperparameters using fivefold cross-validation (GridSearchCV).

In this research, many models were trained on the dataset for almost every ML project before choosing the one that performs the best. However, there is still potential for improvement because there is no certainty that this specific model is the best for the issue at hand. As a result, the aim is to make the model better in whatever manner. These models’ hyperparameters play a crucial role in how well they function: if the correct values are chosen for these hyperparameters, the performance of the model performance can advance considerably. Grid search cross-validation (GridSearchCV) was used to choose the best model for each ML approach. With the parameters that produced the better cross-validation performance, a new model is automatically fitted using this method to the whole training dataset. This method aids in obtaining a more accurate generalization performance estimate. For example, with *k* = 5, the K-fold cross-validation procedure was used. A portion of the data is used in the K-fold cross-validation to test the model and another portion to fit it. The prediction error from Equation (8) is then estimated using cross-validation as follows:

(8) $$CV\left(f\right)=\frac{1}{n}\overset{n}{\underset{i=1}{\sum }}T\left(y_{i},\;f^{-k\left(i\right)}\left(xi\right)\right)$$

where k is the number of subsets, n is the size of the dataset, T is the loss function, and $f^{-k\left(i\right)}$ is the fitted function. The GridSearchCV methodology utilized in this study for model training and hyperparameter selection is shown in Figure 2 as demonstrated in [48].

### 4.5. Model Interpretation with the SHAP Method

In this research, SHAP was applied to the GSC-GBRT model output of the predicted values to explain them. This technique is known as the decoupling of each input parameter’s effect on the Cs of a particular mixture sample. A model of explanation developed by SHAP is expressed as follows:

(9) $$g\left(z^{\prime }\right)=\phi_{0}+\overset{K}{\underset{j=1}{\sum }}\phi_{j}z_{j}^{\prime }$$

where $\phi_{0}$ is the constant if all inputs are missing, $\phi_{j}$ stands for the i-th feature contribution value, and $z^{\prime }\in$ and *K* represent the number of input features. A mechanism is created to determine how much each input information adds to the value that the model generates. The Shaply value is the one that most closely resembles human intuition and fits the three criteria that the additive feature attribution approach should meet (local accuracy, missingness, and consistency) [49]. Shaply values indicate the extent that each predictor (feature) contributes to a machine learning model.

Two models are trained: $f_{S\cup }$ when a particular feature i is included and $f_{S}$ when it is not, to investigate the impact of that feature on the model. The variance in the results obtained from these two models for a given input $x_{S}$ reveals how feature i affected the model. This theoretical idea serves as the foundation for the final calculation of the Shapely value, which represents the contribution of each feature, as the weighted average of all potential differences, as indicated in the following equation:

(10) $$\phi_{j}=\sum_{S\subseteq F\backslash \left\{i\right\}}\frac{\left|S\right|!\left(\left|F\right|-\left|S\right|-1\right)!}{\left|F\right|!}\left[f_{S\cup \left\{i\right\}}\left(x_{S\cup \left\{i\right\}}-f_{S}\left(x_{S}\right)\right)\right]$$

where S represents the set of all features excluding i, F represents the set of all features, and f is the prediction/estimation model.

### 4.6. Performance Metrics

The coefficient of determination (R^2^), as indicated by Equation (11), was used to evaluate the accuracy of the training and testing datasets for each model.

(11) $$R^{2}=1-\frac{\sum_{i=1}^{n}{\left(y_{i}^{obs}-y_{l}^{pre}\right)}^{2}}{\sum_{i=1}^{n}{\left(y_{i}^{obs}-y_{i}^{-obs}\right)}^{2}}$$

where $y_{i}^{pre}$ and $y_{i}^{obs}$ represent the predicted output and the actual outcome (Cs), respectively; n denotes the number of data used in the Cs modeling, and $y_{i}^{-obs}$ is the mean value of the real outcome.

The root mean square error (RMSE) of the algorithm prediction was computed from Equation (12). The comparison between RMSE values can be used to assess the optimization process since a more accurate model has a comparatively lower RMSE value.

(12) $$RMSE=\sqrt{\frac{1}{n}\sum_{i=1}^{n}{\left(y_{i}^{obs}-y_{l}^{pre}\right)}^{2}}$$

Another performance metric used in this study is the variance accounted for (VAF). The following equation was used to compute this metric.

(13) $$\mathrm{VAF}=\left[1-\frac{\mathrm{VAR}\left(y_{i}^{obs}-y_{i}^{pre}\right)}{\mathrm{VAR}\left(y_{i}^{obs}\right)}\right]\times 100$$

When the RMSE, R^2^, and VAR become closer to 0, 1, and 100, respectively, the accuracy of the model prediction increases.

## 5. Dataset Used

Various studies have investigated the Cs of environmentally friendly concrete. As a result, an extensive dataset with 164 experiments on the Cs of eco-friendly concrete incorporating both RA and GGBFS material was recently assembled in reference [50]. The ML model for predicting the concrete Cs was trained and tested using this dataset. Details can be consulted in [50]. According to [39], the data collected from the collected experimental studies consider the effect of both GGBFS and RCA on the Cs of concrete. To unify their results, when collecting the data, specimens with only cube shapes of either 100 mm or 150 mm in length were considered. The authors used Rashid and Mansur’s equation to transform the Cs from a 100 mm cube specimen to the equivalent Cs from a 150 mm cube specimen. In addition, five of the data records had no RA and/or GGBFS in their combination proportions, indicating standard concrete blends.

After being randomly sorted for the aim of constructing models, the gathered data records were divided into two fragments. First, the data records were split into training and testing data with 80% and 20%, respectively. Some relevant key parameters of the dataset utilized in this work is shown in Table 2. The primary input factors that have a significant impact on the concrete Cs were the following ones: water/binder ratio (W/B), curing time (Age), the recycled aggregate percentage from total aggregate in the mixture (RA%), GGBFS material percentage from total binder used in the mixture (GGBFS%), and superplasticizer content (kg). Table 2 presents the lowest, average, median, standard deviation, and maximum values of the input variables for the training and testing sets.

**Figure 3:**
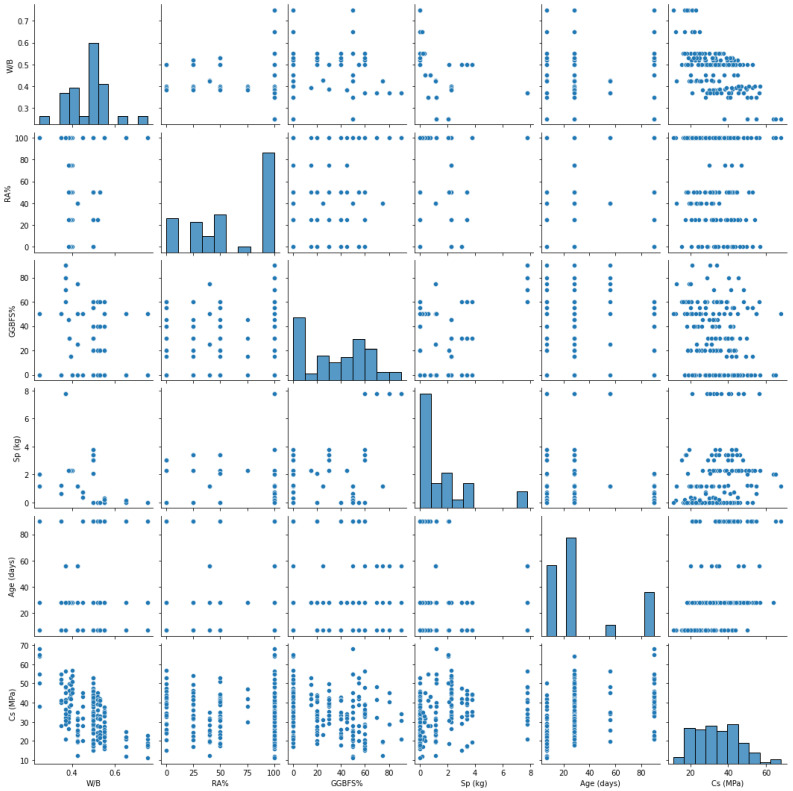
Correlations between the Cs and the primary concrete components in the dataset, as well as their statistical distributions.

Figure 3 displays the relationships and statistical distributions of the Cs and the concrete components. Notably, none of the components are correlated, meaning that all the variables considered to forecast the concrete Cs are independent. Additionally, it can be seen that there is only a weak correlation between the W/B ratio and the Cs.

**Figure 4:**
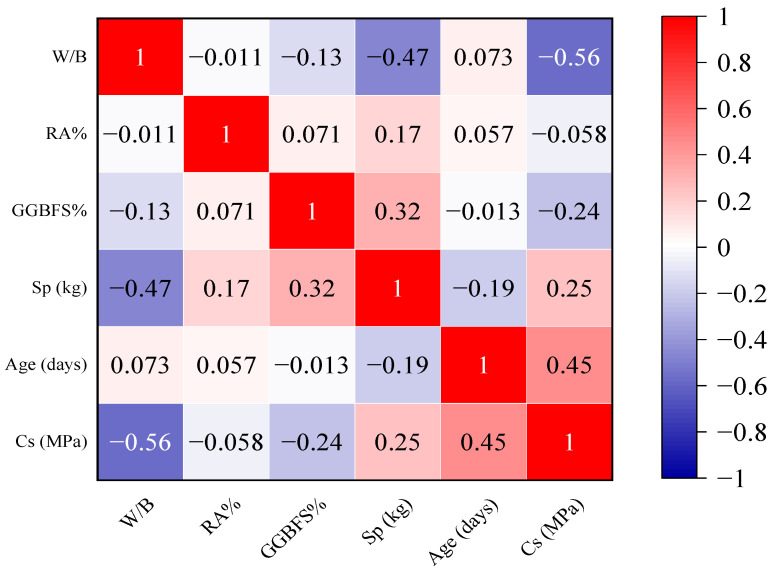
Heatmap showing the correlation between the dataset’s Cs, age, and each eco-friendly concrete component.

A heatmap, as shown in Figure 4, clearly summarizes the association of the entire dataset (containing all of the ratios, weight, percentages, age, and Cs of concrete). It is possible to plot the heatmap in Python by using the Seaborn library. A correlation index near 1 indicates that the features are highly connected. A negative correlation value close to −1 indicates a perfect correlation of two features but moving in opposing directions. In contrast, two uncorrelated features have a correlation index near 0. As it can be noticed in the heatmap, W/B, RA%, and GGBFS% have a negative correlation with the output, while Age and Sp have a positive correlation.

The parameters in Table 2 are utilized to produce environmentally friendly concrete. For completeness, a brief description of each variable is provided below to justify why they were considered concrete components in this study.

The W/B ratio has a significant influence on the concrete strength. Researchers noted that reducing the W/B ratio enhanced the Cs, splitting tensile strength, net flexural strength, and elastic modulus of self-compacting concretes fabricated using RA [51]. Moreover, the 28-day Cs of RCA concretes with a W/B ratio of 0.40 could be increased to be higher than the 28-day Cs of NA concrete with a W/B ratio of 0.50 and be closer to the 28-day Cs of NA concrete with a W/B ratio of 0.45 through the use of fly ash at replacement rates of 15 and 25% by weight of binder in RCA concretes [52]. In addition, the test findings demonstrated that the fly ash contributed more for the Cs at lower W/B ratios than it did in mixes made at higher W/B ratios.

The use of demolition waste as RA to create RAC has been investigated by several researchers [53,54]. However, RAC characteristics are inferior to NCA when porous mortar binds the RA [15]. Furthermore, comparing RCA to NCA, a 30–40% reduction in Cs was noticed [16]. Regardless, Aliabdo et al. [55] found that while replacing coarse aggregate with RA results in a negligible drop in concrete strength, substituting fine recycled aggregate results in a considerable reduction. Therefore, RA% was added as a necessary concrete element in the dataset used in this study.

With the incorporation of RA, Cs tends to drop; however, adding superplasticizers (Sp) can improve the mix compactness, recovering up most of the strength loss [56]. Cs increased when Sp dose was raised, and it was even more significant for Sp with higher water reduction capacities. With the proportion of added Sp, the mix density followed a similar pattern to Cs and slightly decreased towards a higher dosage [56].

Some researchers discovered that the Cs of all self-compacting concrete (SCC) mixes dropped with an increase in RCA for SCC incorporating GGBFS and RCA [57]. GGBFS as a cement replacement reduced the Cs at an early age compared to reference concrete, but at later ages (56 and 90 days), the Cs was equal or higher. The maximum Cs was achieved for SCC mixtures containing 15% GGBFS [57].

The development of durable concrete is determined by its age. When both RCA and GGBFS content are increased, the short-term and long-term Cs of concrete fall [10]. However, the strength growth rate with age increases when both RCA and GGBFS rise. This increase in strength growth rate results from GGBFS latent hydraulic activity and increased hydration of the unhydrated cement particles in RCA [10].

## 6. Model Results

### 6.1. Hyperparameter Optimization: GridSearchCV

Throughout applying the exhaustive parameter search GridSearchCV method, it was possible to find the parameters that matched the predicted model characteristics. Finding parameters with the optimal model estimation accuracy is easier using the Python GridSearchCV function [58]. The ideal GBRT hyperparameter combination was obtained by defining the values and ranges of the GBRT estimation model hyperparameter and using the GridSearchCV function. The learning rate and the n estimators, which stand for the weights given to each estimator and the number of the model weak learners, respectively, are the most crucial hyperparameters for the GBRT model. Additionally, the subsample parameter, which denotes the percentage of data to be utilized for fitting the individual base learners, and the max depth parameter, which defines the complexity of each tree, can both significantly impact the GBRT model ability to predict outcomes. When the following parameters are set: learning_rate = 0.05, max_depth = 4, n_estimators = 1000, subsample = 0.5, the GBRT using the GridSearchCV approach yields high estimate accuracy. The tuning parameters taken into account by GridSearchCV are listed in Table 3.

### 6.2. Comparison of the Prediction Results of the Two Models

**Figure 5:**
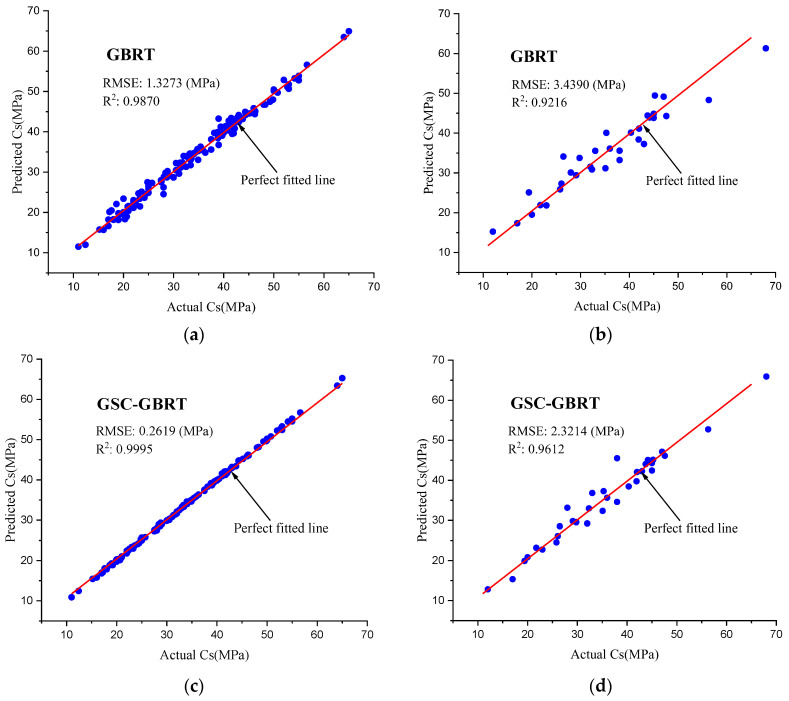
Results of the GSC-GBRT and GBRT models for the testing and training datasets. (**a**) GBRT training set. (**b**) GBRT testing set. (**c**) GSC-GBRT training set. (**d**) GSC-GBRT testing set.

The observed and predicted Cs values of the testing and training datasets are shown in Figure 5 to understand better the performance of the GSC-GBRT. Figure 5 demonstrates that, when compared to the default GBRT predictive model, the Cs outcomes predicted by the GSC-GBRT model were more in line with their observed values. In addition, the prediction accuracy of the GSC-GBRT test set after the hyperparameters’ modification was higher than that of the default GBRT model, according to the optimized hyperparameter findings (see Table 4). For example, the R^2^ and RMSE values for the GSC-GBRT model were 0.9612 and 2.3214, respectively, whereas they were 0.9216 and 3.4390 for the GBRT model. This demonstrates that, for the eco-friendly concrete dataset used for the prediction procedure of the Cs, GSC-GBRT could better match the complicated connection between the component factors influencing the Cs and had superior generalization capacity.

**Figure 6:**
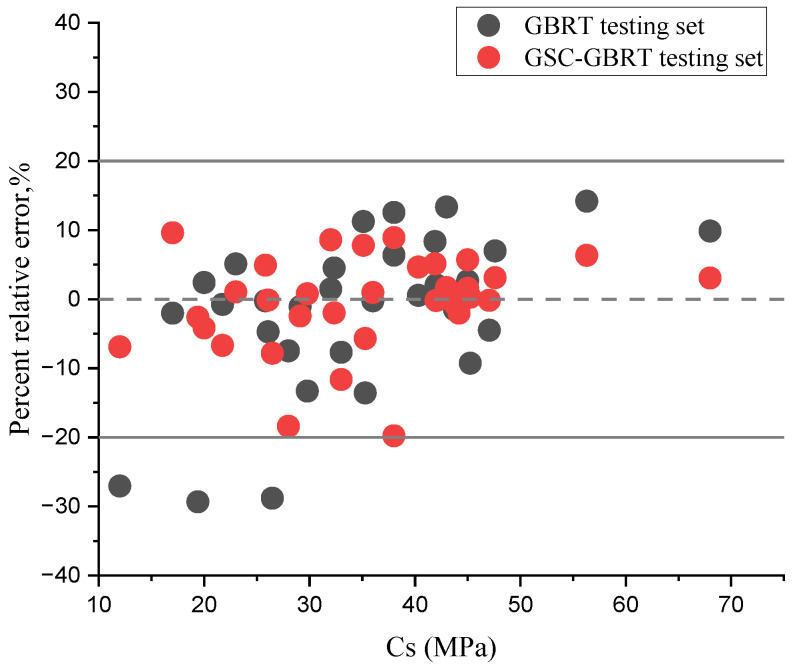
Error distribution for the developed models.

Figure 6 displays the distribution of the relative percentage error between the values of the GBRT and GSC-GBRT models and the Cs of environmentally friendly concrete. Based on this graph, it can be stated that the relative error distributions around the zero-error line for both models during testing are suitable. However, as shown by the reported findings in Figure 6, the GSC-GBRT model appears more capable of making predictions than the GBRT model.

**Figure 7:**
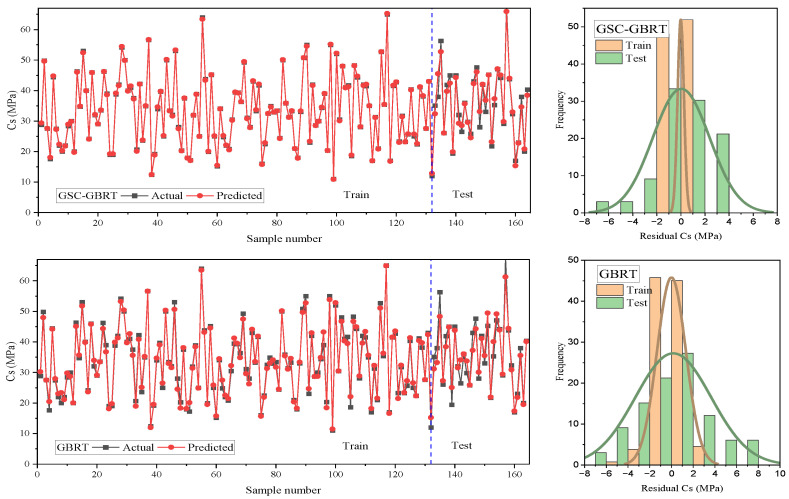
Model errors between targets and predictions for testing set (**a**) GSC-GBRT, (**b**) GBRT.

The results of residuals produced by GSC-GBRT and default GBRT prediction of the Cs of environmentally friendly concrete mixtures at various ages are shown in Figure 7. Each figure shows red dots for the predicted values and black square for measured values. The predicted and measured values of the training set was relatively close for both methods. However, some of the GBRT calculations included inaccuracies exceeding 5 MPa. As it can be noticed, the testing set analysis showed that while GSC-GBRT maintained relatively constant prediction findings, several GBRT predicted points significantly differed from the actual Cs values.

**Figure 8:**
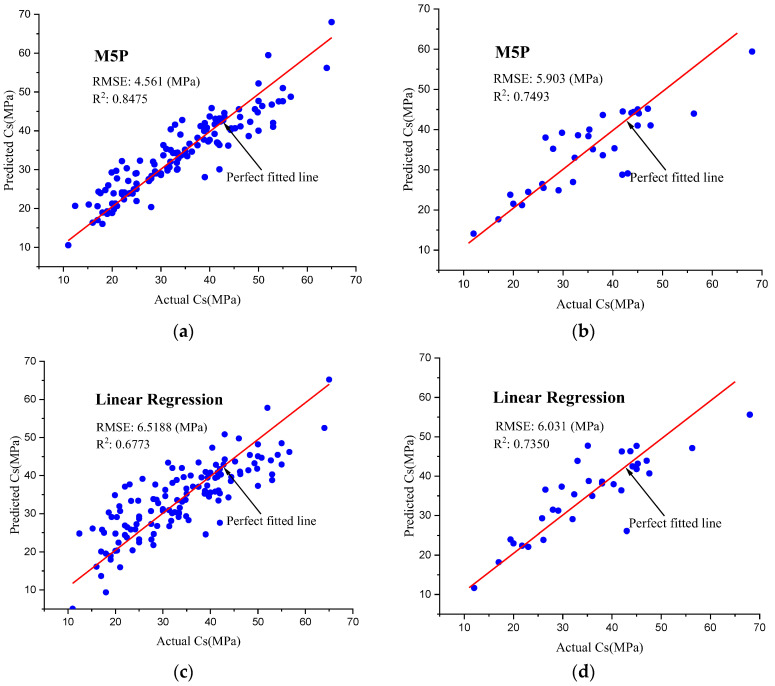
Results of the M5P and linear regression models for the testing and training datasets. (**a**) M5P training set. (**b**) M5P testing set. (**c**) Linear regression training set. (**d**) Linear regression testing set.

The multiple linear regression and M5P models [59,60] are compared with the GSC-GBRT model in order to more accurately depict the accuracy of the last one. Multiple linear regression and M5P were used to obtain the predictions, and an 8:2 dataset division ratio was used. Figure 8 depicts the correlation between the estimated and actual Cs.

Table 5 displays the evaluation results for the test set using multiple linear regression and M5P. It can be shown that the GSC-GBRT ensemble learning model outperforms both of the other models. Multiple weak learners produced by different learning algorithms are combined in ensemble learning. To encourage more accurate predictions, weak learners who perform well are given larger weights, while weak learners who perform poorly are given lower weights. On the test set, the GSC-GBRT ensemble learning model performed the best. When compared to the linear regression, the R^2^ was up 31%, and the RMSE was down 62%. R^2^ increased by 28%, and RMSE dropped by 61% when compared to the M5P model. Overall, the ensemble learning model appears to perform significantly better for the eco-friendly concrete Cs prediction than the traditional machine learning methods.

## 7. Interpretation of the GBRT Model

**Figure 9:**
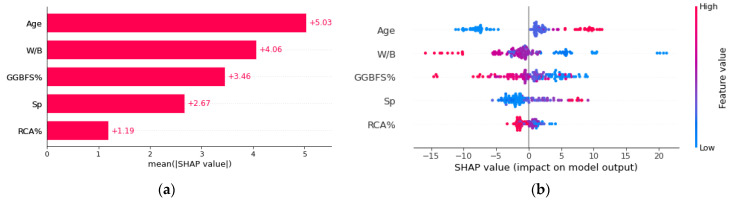
The relative importance of each feature and SHAP summary plot: (**a**) relative importance; (**b**) SHAP summary plot.

In the absence of supporting theories, mathematical computations, or operational processes, the output findings or predictions of ML modeling can be challenging to explain [61]. However, the contributions of input variables can be analyzed using feature importance, sensitivity analysis, or partial dependency analysis in order to evaluate those outputs and comprehend the trained models. Here, the multicollinearity problem and potential synergistic effects of the variables were effectively treated using the SHAP approach. In this section, the “SHapley Additive Explanations” approach [62], combined with the GSC-GBRT model, explains and clarifies the contribution of each input variable to the concrete Cs prediction. According to Figure 9a, which displays the mean absolute SHAP values for each feature in the Cs modeling, the age in the concrete mixture has the highest mean SHAP value among the five input features. The descending order of input variable impact on GSC-GBRT model prediction accuracy is: AGE > W/B > GGBFS% > SP > RCA%.

The input features SHAP values are shown in Figure 9b, with red denoting a high feature value and blue denoting a low feature value. The corresponding feature will benefit the output goal if the feature SHAP value is positive. When the SHAP value is more significant, the influence has a greater impact. For example, the age (red points) in Figure 9b exhibited noticeably high SHAP values, which showed that the higher curing age had a detrimental effect on the prediction of the Cs. It is widely established from Figure 9b that the W/B content, RCA%, and GGBFS% have a negative association with Cs. This is in line with earlier research [8,63].

A web application was developed using the suggested GSC-GBRT model to forecast the Cs of eco-friendly concrete at the Streamlit library. The user can theoretically forecast the Cs of eco-friendly concrete using the trained ML model based on the compiled dataset. W/B, RA%, GGBFS%, superplasticizer (kg), and age (days) are the first parameters the user enters using the online application sliders and radio buttons. In the next step, the concrete Cs in MPa is computed. The parameter ranges provided in the application match the feature ranges in the datasets used for ML training. The Streamlit web application can be accessed at the link given in [64].

## 8. Conclusions

This research proposed a hybrid GSC-GBRT model for the prediction of the Cs of sustainable concrete. The GridSearchCV approach was firstly used to find the optimum parameters, and the optimized model was then used to forecast the Cs. The hybrid GSC-GBRT model obtained higher prediction accuracy and reduced error with R^2^ = 0.9612 and RMSE = 2.3214 when compared to the evaluation metrics of the original GBRT model with R^2^ = 0.9216 and RMSE = 3.4390 for the test set. The suggested GSC-GBRT model surpasses the initial GBRT model in assessment metrics, and it is suggested to be used as a tool for pre-estimating the Cs of concrete using the mix ratio prior to design and mixing.

According to the SHAP-based research, W/B and age are the two input factors that most significantly impact the concrete Cs among the five considered. Age and superplasticizer positively impact the output, and the Cs rises as a result. On the other hand, a rise in W/B, GGBFS%, and RA% causes the Cs to fall. Therefore, designers and engineers can use the significance and contribution of these factors to the output outcomes as a guide. Finally, in Streamlit, a web application for predicting Cs of eco-friendly concrete, was developed. The cloud has been used to deliver the application light version. Any web browser, including mobile ones, can be used to access and use it.

The GSC-GBRT model has several limitations while being competent and acceptable for estimating the Cs of eco-friendly concrete. Firstly, 164 experiments from the literature were used to construct the eco-friendly concrete dataset. The accuracy of the prediction models is significantly influenced by the completeness of the data, quantity, quality, and distribution of the input parameters. As new experimental data become available, the dataset may benefit from being updated. Secondly, like any ML method, the SHAP explanations and GSC-GBRT findings may only apply to the tested input data ranges.

## Figures and Tables

**Table 1:** Various algorithms to predict the Cs of eco-friendly concrete.

Algorithm Used	Data Points	Reference	Year	R^2^	RMSE (MPa)	Replacement Material Used	Limitation
Gene Expression Programming (GEP)	251	[23]	2017	_	7.9	RCA	No GGBFS material included
M5 model tree (M5)	156	[24]	2020	_	8.3	RCA	No GGBFS material included
Multivariate Adaptive Regression Splines (MARS)	156	[24]	2020	_	9.1	RCA	No GGBFS material included
Least Squares Support Vector Regression (LSSVR)	156	[24]	2020	_	7.7	RCA	No GGBFS material included
convolutional neural network (CNN)	74	[25]	2018	_	_	RCA	No GGBFS material included
Multiple nonlinear regression (MNR)	650	[26]	2019	0.027	11.94	RCA	No GGBFS material included
imperialist competitive algorithm-extreme gradient boosting (ICA-XGBoost)	209	[27]	2021	0.983	1.147	RCA	No GGBFS material included
imperialist competitive algorithm—adaptive network-based fuzzy inference system (ICA-ANFIS)	209	[27]	2021	0.940	2.770	RCA	No GGBFS material included
imperialist competitive algorithm-Artificial neural network (ICA-ANN)	209	[27]	2021	0.960	2.225	RCA	No GGBFS material included
imperialist competitive algorithm-Support Vector Regression (ICA-SVR)	209	[27]	2021	0.962	2.149	RCA	No GGBFS material included
Backpropagation neural network models (BPNN)	344	[28]	2020	0.828	6.639	RCA	No GGBFS material included
Random Forest (RF)	453	[21]	2021	0.946	4.958	GGBFS	No RCA material included
ANN	284	[29]	2009	0.981	2.511	GGBFS	No RCA material included
ANFIS	284	[29]	2009	0.968	3.379	GGBFS	No RCA material included
Hybridized multiobjective ANN and a multiobjective slap swarm algorithm (MOSSA)	624	[30]	2020	0.941	2.39	GGBFS	No RCA material included
M5P model tree algorithm	624	[30]	2020	0.883	4.60	GGBFS	No RCA material included
ANN model	269	[22]	2019	0.961	3.332	GGBFS	No RCA material included

**Table 2:** Input and output key parameters for the dataset.

Data Category	Statistics	Sp (kg)	RA%	Age (days)	W/B	GGBFS%	CS (MPa)
Training data	Standard deviation	2.12	38.22	29.52	0.10	26.14	11.52
	Mean	1.57	58.70	34.54	0.48	33.47	33.81
	Median	0.76	50.00	28.00	0.50	30.00	33.30
	Maximum	7.80	100.00	90.00	0.75	90.00	65.00
	Minimum	0.00	0.00	7.00	0.25	0.00	11.00
	Kurtosis	2.56	−1.48	−0.30	1.34	−1.14	−0.49
Testing data	Standard deviation	2.27	38.80	31.27	0.10	24.91	11.89
	Mean	1.71	70.91	35.85	0.46	31.21	35.38
	Median	0.76	100.00	28.00	0.50	40.00	35.28
	Maximum	7.80	100.00	90.00	0.75	80.00	68.00
	Minimum	0.00	0.00	7.00	0.25	0.00	12.00
	Kurtosis	2.73	−0.80	−0.62	1.14	−1.31	0.53

**Table 3:** Hyperparameters for the GBRT model.

Tunning Hyperparameter	Values and Ranges	Optimal Hyperparameters
n Estimators	[100, 500, 1000]	1000
Learning Rate	[0.01, 0.05, 0.1, 0.2]	0.05
Max Depth	[4, 6, 8, 10]	4
Subsample	[0.9, 0.5, 0.2, 0.1]	0.5

**Table 4:** GSC-GBRT and GBRT model performance in predicting the Cs for training and test datasets.

Sets	Model	RMSE	R^2^	VAF(%)
Training	**GSC-GBRT**	**0.2619**	**0.9995**	**99.95**
	GBRT	1.3273	0.9870	98.66
Testing	**GSC-GBRT**	**2.3214**	**0.9612**	**96.07**
	GBRT	3.4390	0.9216	91.40

**Table 5:** GSC-GBRT, linear regression, and M5P models’ performance in predicting Cs for training and test datasets.

Model	RMSE	R^2^	VAF(%)
**GSC-GBRT**	**2.3214**	**0.9612**	**96.07**
Linear regression	6.031	0.735	73.49
M5P	5.903	0.7493	74.87

## Data Availability

Not applicable.
